# Non-coding RNAs as modulators of radioresponse in triple-negative breast cancer: a systematic review

**DOI:** 10.1186/s12929-024-01081-y

**Published:** 2024-10-02

**Authors:** Maria Vitoria Tofolo, Fernanda Costa Brandão Berti, Emanuelle Nunes-Souza, Mayara Oliveira Ruthes, Lucas Freitas Berti, Aline Simoneti Fonseca, Daiane Rosolen, Luciane Regina Cavalli

**Affiliations:** 1 Research Institute Pelé Pequeno Príncipe, Faculdades Pequeno Príncipe, Instituto de Pesquisa Pelé Pequeno Príncipe, Av. Silva Jardim, 1632, Curitiba, 80250-060 Brazil; 2 Department of Mechanical Engineering, Postgraduate Program in Mechanical and Materials Engineering, Universidade Tecnológica Federal do Paraná, Curitiba, 81280-340 Brazil; 3 Department of Oncology, Lombardi Comprenhensive Cancer Center, Washington, DC 20007 USA

**Keywords:** Non-coding RNAs, Radiation, Radiotherapy, Radioresponse, Triple-negative breast cancer

## Abstract

Triple-negative breast cancer (TNBC), characterized by high invasiveness, is associated with poor prognosis and elevated mortality rates. Despite the development of effective therapeutic targets for TNBC, systemic chemotherapy and radiotherapy (RdT) remain prevalent treatment modalities. One notable challenge of RdT is the acquisition of radioresistance, which poses a significant obstacle in achieving optimal treatment response. Compelling evidence implicates non-coding RNAs (ncRNAs), gene expression regulators, in the development of radioresistance. This systematic review focuses on describing the role, association, and/or involvement of ncRNAs in modulating radioresponse in TNBC. In adhrence to the PRISMA guidelines, an extensive and comprehensive search was conducted across four databases using carefully selected entry terms. Following the evaluation of the studies based on predefined inclusion and exclusion criteria, a refined selection of 37 original research articles published up to October 2023 was obtained. In total, 33 different ncRNAs, including lncRNAs, miRNAs, and circRNAs, were identified to be associated with radiation response impacting diverse molecular mechanisms, primarily the regulation of cell death and DNA damage repair. The findings highlighted in this review demonstrate the critical roles and the intricate network of ncRNAs that significantly modulates TNBC's responsiveness to radiation. The understanding of these underlying mechanisms offers potential for the early identification of non-responders and patients prone to radioresistance during RdT, ultimately improving TNBC survival outcomes.

**Graphical Abstract:**

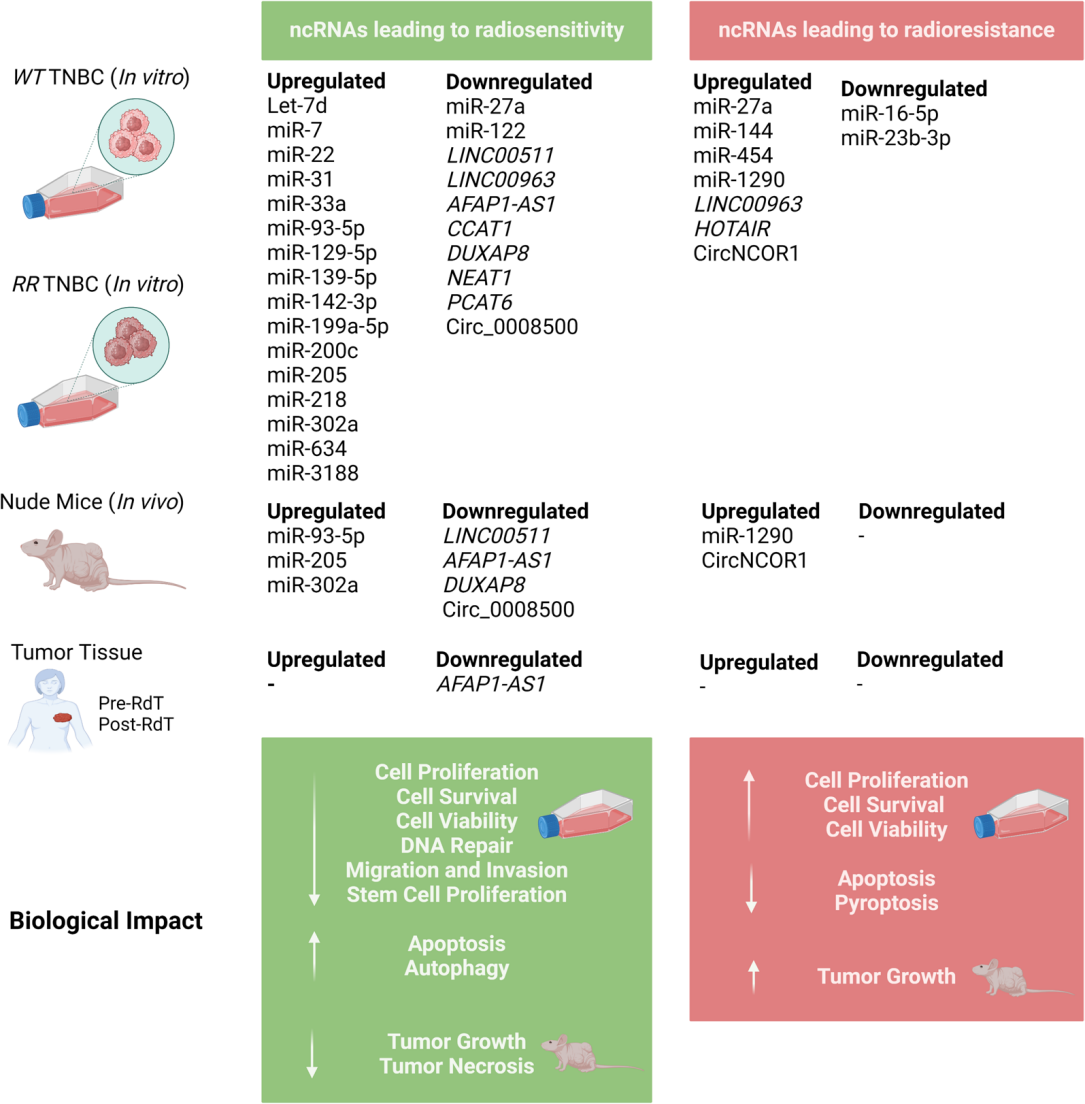

## Background

The acquisition of radioresistance remains a significant challenge in the effective radiotherapy (RdT) treatment in triple-negative breast cancer (TNBC). To the best of our knowledge, there are only few recent reviews exploring the identification and role of non-coding RNAs (ncRNAs) in the modulation of radioresponse in TNBC. However none of them based on a systematic approach such as the one conducted in this review. Considering such gap in the literature and the global burden and clinical aggressiveness of TNBC, our systematic review aims to comprehensively describe the current knowledge on the role, association, and/or involvement of ncRNAs in modulating radioresponse in TNBC. Our current review comprehensively and systematically synthesizes the existing knowledge on ncRNAs in TNBC and radiotherapy, and indicates the potential use of such molecules as promising biomarkers in TNBC patients undergoing radiotherapy.

## Introduction

Breast cancer (BC) is a heterogeneous disease representing the most frequent type and leading cause of death among women worldwide [1]. The triple-negative breast cancer (TNBC) subtype, the most aggressive of the BC subtypes, accounts for approximately 15–20% of all diagnosed BC cases [2]. These tumors are characterized by the lack or lower levels of estrogen receptor (ER), progesterone receptor (PR), and human epidermal growth factor receptor-type 2 (HER2) expression and consequently, therapies targeting these receptors prove ineffective for TNBC treatment [3–5]. Recent advancements have yielded target therapies for TNBC, including the ones based on poly (ADP-ribose) polymerase PARP and immune inhibitors, such as programmed cell death protein-1, PD-1 and its ligand PDL-1 [6]. Nonetheless, only a subset of patients is eligible for these therapies, with the majority still undergoing systemic chemotherapy and radiotherapy.

Radiotherapy (RdT) is widely applied in the oncology practice worldwide [7, 8]. RdT is based in the application of ionizing radiation (IR) to induce DNA damage in tumor cells, thereby inhibiting their ability to proliferate and survive. IR can affect cells directly, promoting DNA damage, such as single-strand-breaks (SSB) and double-strand-breaks (DSB), ultimately leading to genomic instability and programmed cell death, or apoptosis. Additionally, IR can affect cells indirectly by generating reactive oxygen species (ROS), which induce complex DNA lesions that may lead to cell death [9]. TNBC patients typically receive RdT either as a standalone treatment or in combination with other therapeutic modalities. This combined approach aims to enhance treatment effectiveness and prognosis, particularly following breast-conserving surgery [3].

Fundamentally, RdT is expected to be effective across a broad spectrum of tumor cells. However, the inherited heterogeneity of tumor cells and molecular characteristics, coupled with the influence of the surrounding microenvironment, among other variables, can rend the cells with different sensitivity to radiation resulting in distinct treatment outcomes [10–12]. Consequently, the underlying mechanisms of both intrinsic and acquired radioresistance are complex, arising from multiple factors [13–15]. Despite significant advances in understanding the mechanisms that lead to radioresistance, accurately predicting and overcoming these challenges remains elusive [16]. Non-coding RNAs (ncRNAs), a diverse group of untranslated RNAs that includes microRNAs (miRNAs), long non-coding RNAs (lncRNAs), and circular RNAs (circRNAs), have emerged as key players in regulating the resistance of cancer cells to RdT [9, 17, 18]. These molecules can act independently or cooperatively, modulating RdT response within intricate networks of cancer driver gene targets [19–26]. Several studies have reported miRNAs [27–30], lncRNAs [31–33], and circRNA [34] that modulate radiosensitivity/radioresistance in TNBC, highlighting the potential role of these molecules in patient's response to RdT. The primary aim of this systematic review was to search for articles that described the role, association, and/or involvement of ncRNAs in modulating IR response in TNBC in vitro and in vivo models as well as their impact on patients’ treatment response. Following a comprehensive and refined selection a total of 37 articles were obtained, reporting the action of 33 different ncRNAs in modulating the response to radiation in TNBC. The compiled findings support the significant and multifaceted contribution of ncRNAs to RdT response, highlighting their application as clinical biomarkers for the early identification of non-responders and patients that will developed radioresistance during RdT. In addition, these findings hold the promise of enhancing the effectiveness of RdT in TNBC, offering strategies for novel targeted and personalized cancer therapies based on ncRNAs.

## Materials and methods

This review followed the Preferred Reporting Items for Systematic Reviews and Meta-Analyses (PRISMA) guidelines [35, 36]. The review protocol was registered at the International Prospective Register of Systematic Reviews (PROSPERO) database under the identifier CRD42023429498.

### Data sources and literature search strategy

The articles searched were written in English and published until October 31st 2023 in four databases: PubMed (MeSH terms and free terms), Embase (EMTREE search terms), Scopus (Index Terms search terms), and Lilacs (free terms only). Although similar, the entry terms were specific for each database. For PubMed, the search strategy in MeSH terms was: ((Non-coding RNA[Title/Abstract]) OR (ncRNA[Title/Abstract])) OR (MicroRNA[Title/Abstract])) OR (miRNA[Title/Abstract])) OR (Long non-coding RNA[Title/Abstract])) OR (lncRNA[Title/Abstract])) OR (Circular RNA[Title/Abstract])) OR (circRNA[Title/Abstract])) AND (Radiation[Title/Abstract])) OR (Radioresistance[Title/Abstract])) OR (Radiosensitivity[Title/Abstract])) AND (Triple negative breast cancer[Title/Abstract])) OR (Triple negative breast tumor[Title/Abstract]); for Embase, the search was performed by EMTREE: ((‘untranslated rna’:ab,ti OR ‘microrna’:ab,ti OR ‘long untranslated rna’:ab,ti OR ‘circular ribonucleic acid’:ab,ti) AND ‘radiation’:ab,ti OR ‘radiosensitivity’:ab,ti) AND ‘triple negative breast cancer’:ab,ti.; for the Scopus, the search was performed by the use of the following Index Terms: (“Non-coding RNA” OR “ncRNA” OR “microRNA” OR “miRNA” OR “Long non-coding RNA” OR “lncRNA” OR “Circular RNA” OR “circRNA”) AND Index Terms (“Radiation” OR “Radioresistance” OR “Radiosensitivity”) AND Index Terms (“Triple negative breast cancer”); and finally for Lilacs, free terms were used: ((Non-coding RNA) OR (ncRNA) OR (microRNA) OR (miRNA) OR (long non-coding RNA) OR (lncRNA) OR (circular RNA) OR (circRNA)) AND ((radiation) OR (radioresistance) OR (radiosensitivity)) AND (Triple negative breast cancer). In addition, the “free terms” search was used for all databases, with the terms: “Non-coding RNAs” OR “microRNAs” OR “lncRNAs” OR “circRNAs” AND “Radiation” AND “Triple negative breast cancer” OR “Breast cancer”. The descriptor “Breast cancer” was added in this final search strategy to obtain articles that did not explicitly use the term “Triple negative breast cancer” but included TNBC clinical samples and/or cell lines in their experimental approaches. For the extraction of duplicated articles, the Rayyan program was used (https://www.rayyan.ai).

Additionally, a bibliometric analysis (using the R package Bibliometrix) [37] was conducted to validate the accuracy of the search strategy, which entailed the selection of the most appropriate keywords/descriptors for each database to identify the most suitable articles for subsequent analysis. This analysis was performed exclusively for the PubMed and SCOPUS databases, considering that the Bibliometrix package does not currently support bibliographic data from the Embase and Lilacs databases.

### Study selection and eligibility criteria

Two reviewers independently assessed and selected the studies according to the established inclusion and exclusion criteria. In cases of discrepancies regarding article inclusion, a third reviewer made the final decision. Inclusion criteria comprised: (1) the involvement of a ncRNA (miRNA, lncRNA or circRNA,) in response to IR (radioresistance or radiosensitivity) in TNBC; (2) peer-reviewed articles written in English. Exclusion criteria comprised: (1) non-original articles (reviews), editorials, letters from editors, book chapters, unpublished or non-peer-reviewed studies; (2) articles that did not assess ncRNAs; (3) articles on ncRNAs that did not report on the role, association, and/or involvement of ncRNAs in modulating IR response, or that did not evaluate response to IR, or that only assessed the response to chemotherapy or other type of treatment, while briefly mentioning RdT as a treatment approach; (4) articles that did not include TNBC clinical samples or TNBC in vitro and in vivo models.

### Data extraction

After the selection and eligibility assessment of the studies, two reviewers extracted the following information independently: risk of bias, year of publication, names of authors, country of origin, title, study aim, sample source (patients’ clinical samples, in vitro and in vivo tumor models), type of methodology. For each ncRNA reported, the description of the main results, and role in modulating IR response were considered.

### Quality assessment and risk of *bias*

The Quality in Prognosis Studies (QUIPS) tool was used to assess the quality of the studies and the risk of bias, evaluating the studies in the following six categories: study participation, study attrition, outcome measurement, study confounding, and statistical analysis and reporting [38]. The articles were assessed for quality according to the following classification: high quality (+ + +), with little or no risk of bias; acceptable (+ +), with moderate risk of bias; and low quality ( ±), with a high risk of bias. Based on this classification, the articles received a general evaluation as low, moderate, or high risk of bias. Low quality articles were excluded.

## Results and discussion

### Qualitative synthesis analysis of the articles in adherence to the PRISMA guidelines

**Fig. 1:**
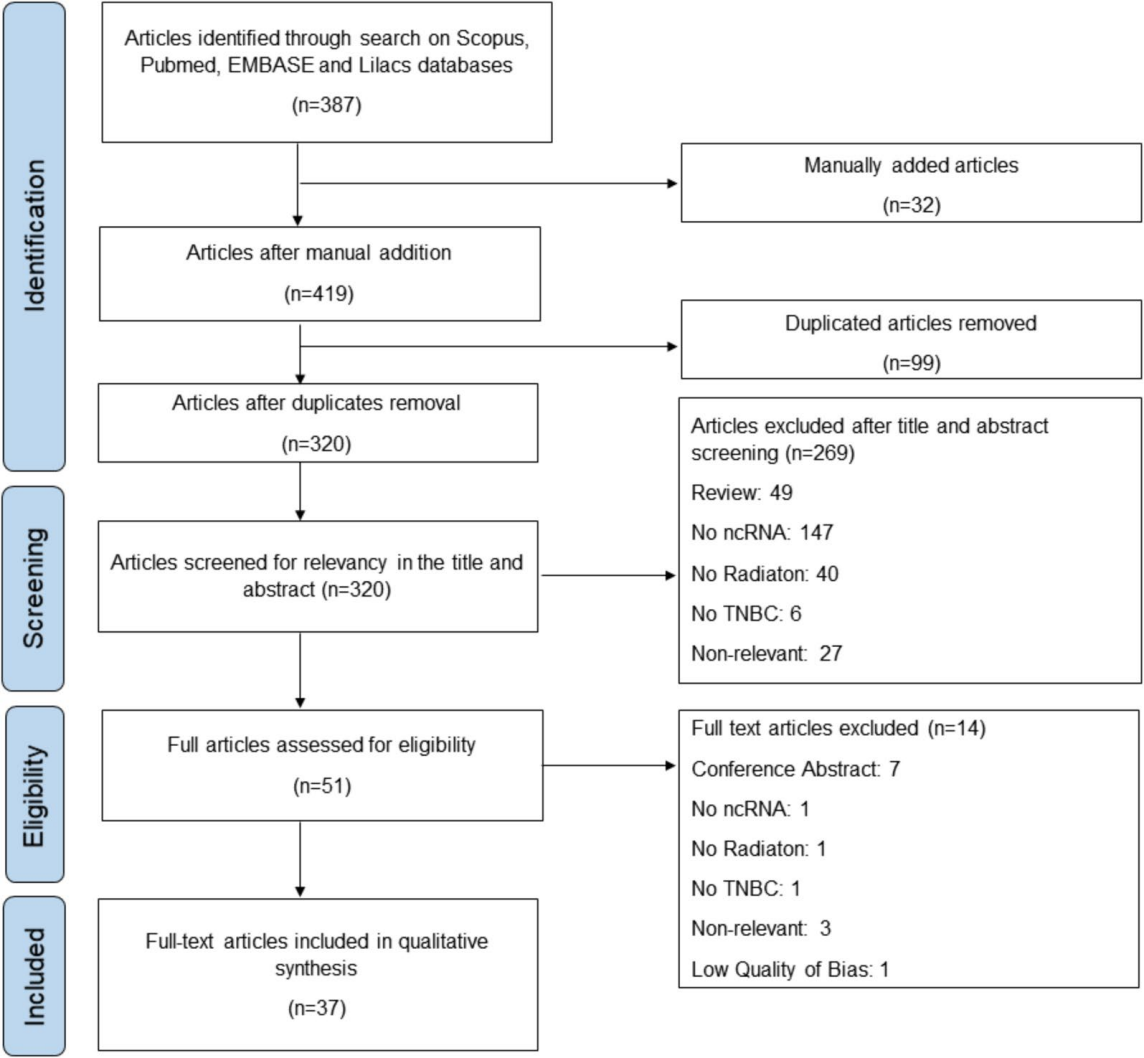
PRISMA Flow Diagram. Flow diagram of the study identification and selection process, following Preferred Reporting Items for Systematic Reviews and Meta-Analyses (PRISMA) guidelines

A total of 387 articles were compiled from all the searched databases, supplemented by additional 32 articles manually added through the free terms search strategy. As a result, 419 articles were considered for further assessment. Following the removal of duplicates (n = 99), 320 articles remained for subsequent assessment based on the established inclusion and exclusion criteria, undergoing initial screening based on abstract and title evaluation. Finally, 51 articles were screened for relevance and fully read. This analysis resulted in 38 full-text articles selected for qualitative analysis, using the six categories of the QUIPS tool [38]. As a result, 37 articles presented a low risk of bias and one a high risk of bias.This article was excluded considering that the outcome measurement of IR was not properly assessed (Fig. 1).

### General characteristics of the selected articles

The 37 original articles selected were published between June 2011 and September 2023. The information on the selected articles is shown in Table 1. The articles were published by research groups from eight different countries, with China presenting the highest number of publications (n = 24 articles), followed by South Korea (n = 3), USA (n = 3), Germany (n = 3), Iran (n = 1), Mexico (n = 1), Australia (n = 1), and Taiwan (n = 1). A total of 33 ncRNAs were described in the selected articles, of which 27 were miRNAs, nine lncRNAs, and two circRNAs.

**Table 1:** Comprehensive analysis and main characteristics of the thirty-seven selected articles

ncRNAs	Samples		ncRNA DE levels	IR dosage (Gy)	Assay(s)—IR effect	Refs.
	TNBC	Controls				
*miRNAs*						
*Let-7d*	*WT*–MDA-MB-231, HS587-T	*WT*–MCF-7; ZR75-1, BT-20	Down	0, 2, 4, 6, 8	Sphere formation	[41]
*miR-7*	*WT*–MDA-MB-468	–	–	0, 2, 4, 6, 8	Colony formation	[27]
*miR-16-5p*	*WT*–MDA-MB-231	*WT*–T47D	Down	0, 2, 4, 6, 8, 10	Cell viability (MTT)	[27]
*miR-22*	*WT*–MDA-MB-231	*WT*–MCF-10A	Down	0, 2, 4, 6, 8 6	Colony formation DNA damage (γ-H2AX)	[40]
*miR-23b-3p*	*WT*–MDA-MB-231	*WT*–T47D	Down	0, 2, 4, 6, 8, 10	Cell viability (MTT)	[27]
*miR-27a*	*WT*–MDA-MB-231, MDA-MB-435	*WT*–MCF-10A	Up	0, 8 0, 8	Cell viability (CCK-8) Apoptosis (Caspase-3)	[28]
*miR-27a*	*WT*–MDA-MB-231, MDA-MB-468	–	–	30, 50, 100 30, 50, 100	Cell Viability (MTT) Apoptosis (Caspase-Glo3/7)	[70]
*miR-31*	*WT*–MDA-MB-231	–	–	5	Cell Viability (CellTiter)	[62]
*miR-33a*	*WT*–MDA-MB-231, SUM159 *WT*–SUM149	*WT* – KPL4 *WT* – KPL4	Up Down	0, 2, 4, 6	Colony formation	[41]
*miR-93-5p*	*W* –MDA-MB-231, MDA-MB-468	–	–	0, 2 0, 2 0, 2	Migration Cell viability (CCK-8) Apoptosis (Annexin V-FITC/PI)	[29]
*miR-93-5p*	Tumor Xenograft model – MDA-MB-231	–	–	FD -12	Histopathological analysis	[55]
*miR-122*	*RR*–MDA-MB-231 *RR*–MDA-MB-231	*WT* – MDA-MB-231 *RR*-MCF-7	Up Up	0, 2, 4, 6, 8 4	Cell viability (MTT) Colony formation	[48]
*miR-129-5p*	*WT*–MDA-MB-231	*WT*–MCF-10A	Down	0, 2, 4, 6, 8 0, 6 0, 6	Colony formation Autophagy (GFP-LC3) Apoptosis (Caspase-3)	[42]
*miR-139-5p*	*WT*–MDA-MB-157, MDA-MB-231, MDA-MB-453, MDA-MB-468, BT-20, HCC1937	–	–	0, 6	Cell survival (MTS)	[67]
*miR-142-3p*	*WT–*MDA-MB-468, HCC1806	–	–	0, 2, 4, 6	Colony formation	[68]
*miR-144*	*WT–*MDA-MB-231, SKBR3	–	–	0, 30, 50, 100 0, 0.5, 2, 5	Cell survival (WST-1) Apoptosis (Caspase-3/-7)	[71]
*miR-199a-5p*	*WT–*MDA-MB-231	Control group^1^	Up	0, 2, 4, 6 8	Cell viability (CCK-8) Autophagy (GFP-LC3)	[49]
*miR-200c*	*WT* – MDA-MB-231, BT549	*WT*–MCF-10A	Down	0, 2, 4, 6, 8 0, 2, 4, 6, 8	Colony formation Cell proliferation	[30]
*miR-200c*	*WT* – MDA-MB-468	–	–	0, 2, 4, 6, 8 0, 2	Colony formation DNA damage (γ-H2AX)	[58]
*miR-200c*	*WT –* MDA-MB-231, BT549	*WT –* MCF-10A	Down	0, 2, 4, 6, 8 0, 6	Colony formation DNA damage (γ-H2AX)	[43]
*miR-205*	*RR* – SUM159 (SUM159-P2) *WT* – MDA-MB-231 Tumor Xenograft model – SUM159-P2	*WT* – SUM159 (SUM159-P0) *_* *_*	Up _ _	0, 2, 4, 6 SD – 15	Colony formation Tumor Volume	[50]
*miR-218*	*WT* – MDA-MB-468	*WT* – MCF-7	Down	2	Colony formation	[46]
*miR-302a*	*WT* – MDA-MB-231, SKBR3 *RR* – MDA-MB-231 Tumor Xenograft model – *RR* – MDA-MB-231	*WT* – MCF-7 *WT* – MDA-MB-231 _	Up Up _	0, 2, 4, 6, 8, 10 SD – 5	Colony formation Tumor Volume	[51]
*miR-454*	*WT* – MDA-MB-231, MDA-MB-468	–	–	0, 20, 40, 60 0, 5, 10, 20	Cell proliferation Apoptosis assay (Caspase-Glo3/7)	[72]
*miR-634*	*RR* – MDA-MB-231	*WT* – MDA-MB-231	Up	0, 2, 4, 6, 8 4	Cell viability (MTT) Apoptosis (Annexin V-FITC/PI)	[52]
*miR-1290*	*RR* – MDA-MB-231 *RR*-Tissues samples	*WT* – MDA-MB-231 *RS*-Tissues samples	Down Down	4 4 SD – 8	Cell viability (MTT) Colony formation Tumor Volume	[45]
*miR-3188*	*WT*-MDA-MB-231, HCC1954	*–*	–	7.5	Cell viability (MTT)	[63]
*lncRNAs*						
*LINC00511*	*WT* – MDA-MB-231, MDA-MB-436	*WT*-MCF-7	Up	0, 2, 4, 6, 8, 10 0, 2, 4, 6, 8, 10 SD-10	Cell viability (MTT) Apoptosis (Annexin V) Tumor Volume	[53]
*LINC00963*	*WT* – MDA-MB-231	*WT* – MCF-12A	Up	0, 2, 6, 10 0, 6 0, 6	Colony formation ROS detection DNA damage (γ-H2AX)	[31]
*LINC00963*	*RR* – MDA-MB-231 *WT* – MDA-MB-231, SKBR3	–	–	4, 8 0, 2, 4, 8, 12 2, 8	Colony formation Cell viability (CellTiter-Glo) Apoptosis	[56]
*AFAP1-AS1*	*RR* – MDA-MB-231 *RR* – Tissues samples Tumor Xenograft model – *RR* – MDA-MB-231	*WT* – MDA-MB-231 *RS* – Tissues samples *_*	Down Down _	0, 2, 4, 6, 8, 10 0, 6, 10 0, 6, 10 0, 6, 10 FD – 10	Cell survival Apoptosis (Annexin V) Migration Invasion Tumor Volume	[32]
*CCAT1*	*WT* – MDA-MB-231	Control group^1^	_	0, 2, 4, 6, 8 6	Colony formation Apoptosis (Caspase-3)	[61]
*DUXAP8*	*WT* – MDA-MB-231, *BT*-549 Tumor Xenograft model – MDA-MB-231	*WT* – MCF-10A *_*	Up _	0, 4, 8 0, 4, 8 SD – 8	Cell viability (CCK-8) Apoptosis (Annexin V) Tumor Volume	[46]
*HOTAIR*	*WT*-MDA-MB-231	–	–	0, 15, 20, 25 0, 6	Cell viability (CCK-8) Colony formation	[33]
*NEAT1*	*RR*-MDA-MB-231	*WT*-MDA-MB-231	_	0, 2, 4 *RR-*Cell line	Colony formation Sphere formation	[57]
*PCAT6*	*WT*–MDA-MB-231, MDA-MB-468 *RR*–MDA-MB-231, *RR*- MDA-MB-468	*WT*-MCF-10A *_*	Up _	0, 2, 4, 6, 8 4 4	Colony formation Cell viability (CCK-8) Apoptosis (Annexin V)	[47]
*circRNAs*						
*Circ_0008500*	*WT*–MDA-MB-468 Tumor Xenograft model – MDA-MB-468	*WT* – MCF-10A *_*	Up _	0, 4 0, 4 0, 4 FD–4	Colony formation Apoptosis (Annexin V) Cell proliferation (EdU) Tumor formation	[34]
*CircNCOR1*	*WT* – MDA-MB-231, BT549 Tumor Xenograft model – MDA-MB-231	_ _	Up _	0, 2, 4, 6 0, 2, 4, 6 0, 6 FD—6	Colony formation Apoptosis (Annexin V) Cell viability (CCK-8) Histopathological analysis	[54]

*DE,* Differentiated expressed ncRNAs in the *TNBC* cells compared to controls; *NcRNAs,* non-coding RNAs; *TNBC,* Triple-negative breast cancer; IR, Ionizing radiation; *Ref,* reference; *Gy*,grays; ^1^Control group used but not specified in the article;–, information not available; *WT,* Wild type; *RR*, Radioresistant; RS, Radiosensitive; Down, downregulated compared with control samples; Up, upregulated compared with control samples

One of the aims of this systematic review was to include studies that identified and described the function of these ncRNAs in modulating the IR response in TNBC cells. However, in some studies the reported expression of a given ncRNA was determined by comparing its expression in the TNBC samples with controls, not necessarily assessing changes in expression in the TNBC samples pre- and post- IR administration. In such cases, the subsequent modulation of the ncRNAs expression was evaluated in experimental assays to determine its impact on the response to various doses of IR. For example, the ncRNAs let-7d, miR-16-5p, miR-23b-5p, miR-22, miR-33a, miR-129-5p, miR-200c, miR-218, miR-1290, and *AFAP1-AS1* were downregulated in the parental TNBC cell lines compared to controls [27, 30, 32, 39–45]. Conversely, miR-27a, miR-33a, miR-122, miR-199a-5p, miR-205, miR-302a, miR-634, *LINC00511*, *LINC00963*, *DUXAP8*, *PCAT6*, Circ_0008500 (has_circ_0008500), and CircNCOR1 (hsa_circ_0042174) were upregulated in the parental TNBC cell lines compared to controls [28, 31, 34, 41, 46–54]

Further, the original articles were also evaluated based on the experimental assays and IR dosages used to determine the effects of IR on TNBC cells. A total of 17 articles used the cell viability assay as the primary methodology for assessing the effects of IR. Other assays used included: clonogenic or colony formation (n = 21), tumor volume measurement (n = 6), cell survival (n = 3), cell proliferation (n = 3), sphere formation (n = 2), ROS generation (n = 1), and tumor formation (n = 1) assays. Other articles determined the impact of IR and the role of the ncRNAs evaluating: apoptosis (n = 15), DNA damage (n = 4), autophagy (n = 2), cell migration (n = 2) and invasion (n = 1).

The studies describing the role of miR-93-5p, miR-205, miR-302a, *AFAP1-AS1*, *DUXAP8*, Circ_0008500, and CircNCOR1 also used in vivo TNBC models, specifically tumor xenografts, to evaluate their role in modulating the response to radiation [32, 34, 45, 46, 50, 51, 54, 55]. These studies included the analysis of tumor volume and weight, and histopathology. Only two studies were conducted in TNBC clinical samples [32, 45]. In these studies, the expression of *AFAP1-AS1* [32] and miR-1290 [45] was evaluated in the tissue samples in relation to the response to RdT. Another interesting approach employed in nine studies [32, 45, 47, 48, 50–52, 56, 57] involved the development of radioresistant (RR) cell lines. The most used radioresistant cell line was the RR-MDA-MB-231 (in eight studies); the RR-MDA-MB 468 and RR-SUM159 cell lines were used in one study each [50]. Regarding the IR dosage, 34 studies used the dose below 10 Gy (with different fractionated dose ratios), while 5 studies used doses above 10 Gy. In studies involving animal models, a higher dosage of IR was applied, ranging between 4–15 Gy, either delivered as a single dose (n = 5) or as a fractionated controlled dosage (n = 4). The application of fractionated doses is consisted with RdT protocols typically applied for TNBC in clinical practice [32, 34, 45, 46, 50, 51, 53–55].

The information above is described for each study in Table 1.

### NcRNAs modulating IR response in TNBC

**Fig. 2:**
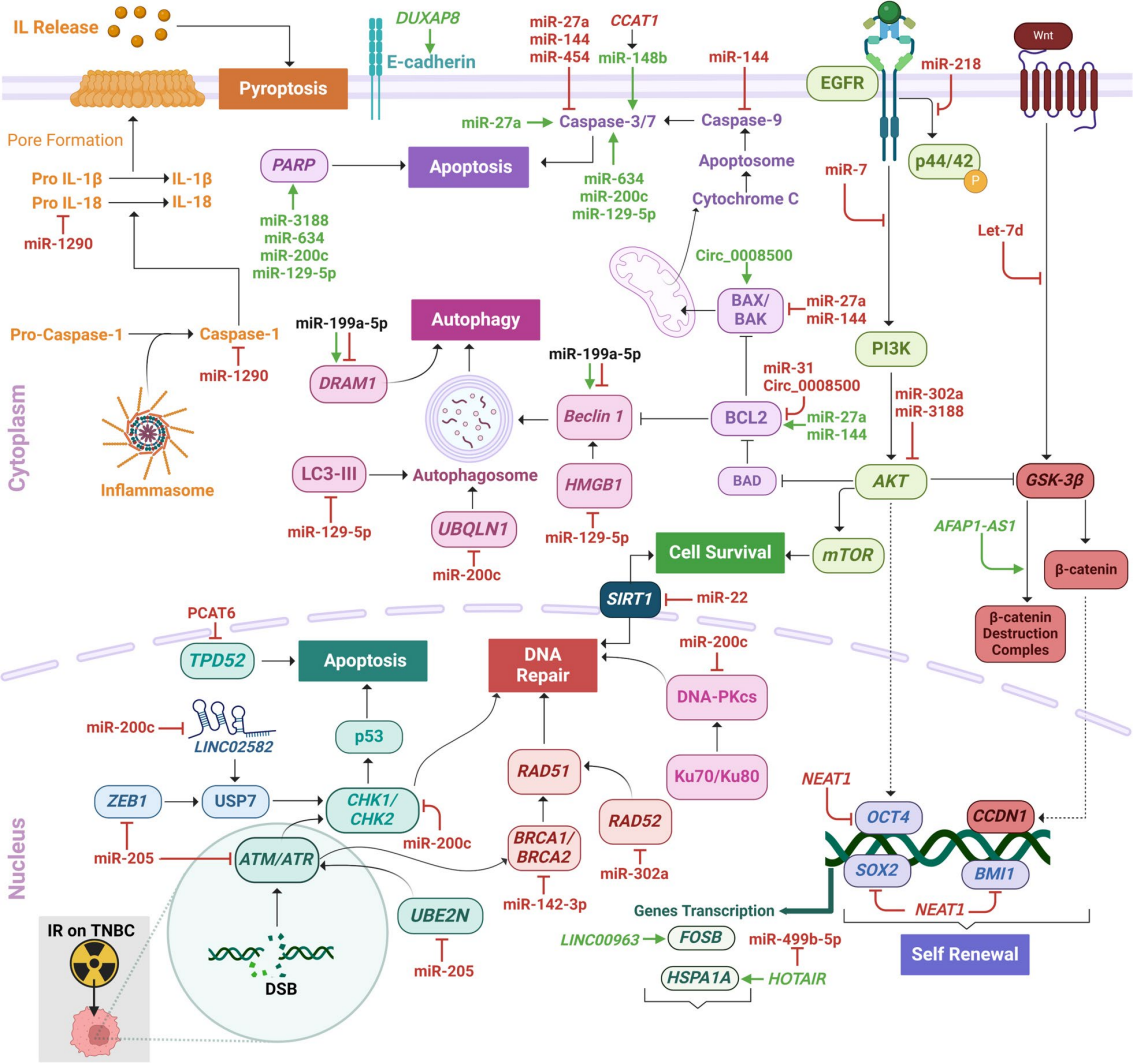
NcRNAs modulating IR response on TNBC irradiated cell lines and corresponding mechanisms. NcRNAs in green and red indicate action on radiosensitivity and radioresistance, respectively. NcRNAs in black present both radiosensitivity and radioresistance action. Image created using *BioRender*

The role, association, or involvement of the ncRNAs in modulating the IR response in the TNBC are summarized in Fig. 2 and Tables 2, 3. Among the 37 selected studies, 29 described the ncRNAs in the modulation of radiosensitivity (Table 2), and nine of radioresistance (Table 3). Among the studies describing the involvement of ncRNAs in modulating radiosensitivity, 21 highlighted the involvement of 18 distinct miRNAs, seven the involvement of seven distinct lncRNAs, and one the involvement of one circRNA. For the modulation of radioresistance, miRNAs were also the most reported ncRNAs, with five studies describing the role of six different miRNAs, followed by two studies on lncRNAs, and one study on circRNA. Gain- and loss-of-function strategies were employed to manipulate the levels of ncRNAs expression and determine their impact on the modulatiion of response to IR. The ectopic expression of the ncRNAs was the most common strategy used (n = 26 studies), followed by expression inhibition (n = 11).

**Table 2:** NcRNAs associated with radiosensitivity on TNBC-expression levels, mechanisms of action and biological impact

ncRNAs	Manipulated expression level	Mechanism of Action	Biological Impact	Refs.
*MiRNAs*				
*Let-7d*	Overexpression	↓Cyclin D1/Akt1/Wnt1 pathway (↓ CCND1)	↓Self-renewal ability ↓ Stem cells population	[39]
*miR-7*	Overexpression	↓EGFR pathway (↓ p-EGRF; ↓ p-Akt; ↓ p-ERK; ↓ p-STAT3)	↓Cell survival	[69]
*miR-22*	Overexpression	↓*SIRT1* (SIRT1)	↓Cell Survival ↓ DNA Repair	[40]
*miR-27a*	Inhibition	↑*CDC27* (CDC27) ↑ Caspase-3	↓Cell Proliferation ↑ Apoptosis	[28]
*miR-31*	Overexpression	↓*PRKCE* (PRKCE) ↓ *BCL2* (BCL2)	↓Cell Viability ↑ Apoptosis	[62]
*miR-*33a	Inhibitor Overexpression	↑ABCA1 (↑ HDL)	↓Cell Survival	[41]
*miR-93-5p*	Overexpression	–	↓Migration ↓ Cell Viability ↑ Apoptosis	[29]
*miR-93-5p*	Overexpression	↓EphA4/NF-Kb pathway	↓Tumor Formation ↑ Tumor Necrosis	[55]
*miR-122*	Inhibition	↑*ZNF304* (ZNF304) ↑ *ZNF611* (ZNF611) ↓ *RIPK1* (RIPK1) ↓ *DUSP8* (DUSP8)	↓Cell Proliferation ↓ Cell Survival	[48]
*miR-129-5p*	Overexpression	↓*HMGB1* (HMGB1) ↓ LC3II ↓ p62 ↑ Caspase-3 ↑ c-PARP	↓Cell Survival ↓ Autophagy ↑ Apoptosis	[42]
*miR-139-5p*	Overexpression	↓DDR pathways (Mutations: *TP53; CDKN2A; PTEN; ATM; EP300; BRAF; KRAS; BRCA1; RB1; SCAI*)	↓Cell Survival ↓ DNA repair	[67]
*miR-142-3p*	Overexpression	↓*BRCA1* (BRCA1) ↓ *BRCA2* (BRCA2) ↓ *BOD1* (BOD1) ↓ *KLF4* (KLF4)	↓Cell Survival ↓ DNA repair ↓ Stem cells population	[68]
*miR-199a-5p*	Overexpression	↑LC3-I ↑ LC3-II ↑ *DRAM1* ↑ *Beclin1*	↓Cell Viability ↑ Autophagy	[49]
*miR-200c*	Overexpression	↓*UBQLN1* (UBQLN1) ↓ LC3II ↑ p62 ↑ Caspase-3 ↑ c-PARP	↓Cell Survival ↓ Autophagy ↑ Apoptosis	[30]
*miR-200c*	Overexpression	↓NHEJ pathway (↓ p-DNA-PKs)	↑Cell Survival ↑ DNA Repair	[58]
*miR-200c*	Overexpression	↓*LINC02582* (↓ *USP7* (USP7)*;* ↓ *CHK1* (CHK1))	↑Cell Survival ↑ DNA Repair	[43]
*miR-205*	Overexpression	↓HR pathway (↓* ATM;* ↓ *ZEB1;* ↓ *UBE2N* (UBC13))	↑Cell Survival ↓ Tumor Growth	[50]
*miR-218*	Overexpression	↓*EGFR* (EGFR) (↓ p44/42 MAPK signaling)	↓Cell Survival	[44]
*miR-302a*	Overexpression	↓*AKT1* (AKT1) ↓ *RAD52* (RAD52)	↓Cell Survival ↓ Tumor Growth	[51]
*miR-634*	Overexpression	↑Caspase-3 ↑ PARP	↓Cell Viability ↑ Apoptosis	[52]
*miR-3188*	Overexpression	↓mTORC2 pathway (↓ Rictor; ↓ p-AKT, ↑ PARP)	↓Cell Viability	[63]
*LncRNAs*				
*LINC00511*	Inhibition	↑*STXBP4* (STXBP4) (Mediated by ↓ miR-185)	↓Cell Viability ↓ Apoptosis ↓ Tumor Growth	[53]
*LINC00963*	Inhibition	–	↓Cell Survival ↓ DNA Repair ↓ ROS	[31]
*AFAP1-AS1*	Inhibition	↑ p-GSK3β (↑ β-catenin destruction complex)	↓Cell Survival ↓ Migration ↓ Invasion ↓ Apoptosis ↓ Tumor Growth	[32]
*CCAT1*	Inhibition	↑Caspase-3 (Mediated by ↑ miR-148b)	↓Cell Survival ↓ Apoptosis	[61]
*DUXAP8*	Inhibition	↑E-cadherin ↑ RHOB	↓Cell Survival ↑ Apoptosis ↓ Tumor Volume	[46]
*NEAT1*	Inhibition	↓ *BMI1* ↓*OCT4* ↓*SOX2*	↓Cell Survival ↓ Stem cells population	[57]
*PCAT6*	Inhibition	↓*TPD52*	↓Cell Viability ↓ Cell Proliferation ↑ Apoptosis	[47]
*CircRNAs*				
*Circ_0008500*	Inhibition	↑*BAX*; ↓ *BCL2* (Mediated by ↓ miR-758-3p/PFN2 axis)	↓Cell Proliferation ↑ Apoptosis ↓ Tumor Growth	[34]

NcRNAs, non-coding RNAs; Ref, reference; ↓, downregulation; ↑, upregulation; ↑ Increase; ↓ Decrease; –, information not available

**Table 3:** NcRNAs associated with radioresistance on TNBC-expression levels, mechanisms of action and biological impact

ncRNAs	Manipulated expression level	Mechanism of Action	Biological Impact	Refs.
*MiRNAs*				
*miR-16-5p*	_	_	↓Cell Viability	[27]
*miR-23b-3p*	_	_	↓Cell Viability	[27]
*miR-27a*	Overexpression	Caspase-3/-7 ↓ BAX ↑ BCL2	↑Cell Viability ↓ Apoptosis	[70]
*miR-144*	Overexpression	↓Caspas-3/-7/-9 ↓ BAX ↑ BCL2	↑Cell Survival ↑ Apoptosis	[71]
*miR-454*	Overexpression	↓Caspase-3/-7	↑Cell Proliferation ↑ Apoptosis	[72]
*miR-1290*	Overexpression	↓*NLRP3* (NLRP3) (↓ IL-18, ↓ IL-1β, ↓ ACS and ↓ Caspase-1)	↑Cell Viability ↑ Cell Proliferation ↑ Tumor Growth ↓ Pyroptosis	[45]
*LncRNAs*				
*LINC00963*	Overexpression	↑*FOSB* ↑ *UBE3C* (UBE3C) ↓ *TP73*	↑Cell Proliferation ↑ Cell Viability ↓ Apoptosis	[56]
*HOTAIR*	Overexpression	↑*HSPA1A* (Mediated by ↓ miR-499b-5p)	↑Cell Survival ↑ Cell Proliferation	[33]
*CircRNAs*				
*CircNCOR1*	Overexpression	↑*CDK2* (CDK2) (Mediated by ↓ miR-638)	↑Cell Proliferation ↑ Cell Viability ↓ Apoptosis	[54]

NcRNAs, non-coding RNAs; Ref, reference; ↓, downregulation; ↑, upregulation; ↑ Increase; ↓ Decrease; –,information not available

#### NcRNAs modulating radiosensitivity

Most of the ncRNAs modulating radiosensitivity were miRNAs (Table 2). Among them, miR-200c was the most cited, reported in three studies [30, 43, 58]. The mechanisms of action attributed to this miRNA, involved the regulation of cell survival, particularly autophagy and apoptosis, and DNA damage repair. Apoptosis and autophagy were modulated by the ectopic expression (overexpression) of miR-200c resulting in the downregulation of the UBQLN1 and LC311 proteins (involved in several aspects of autophagy) and the upregulation of several proteins associated with apoptosis. These expression changes led to the decrease of autophagy and increase of apoptosis [30]. On the other hand, its action on DNA damage repair pathways was evidenced by the γ-H2AX foci formation, along with the downregulation of the phosphorylated DNA-dependent protein kinase catalytic subunit (DNA-PKcs) [58]. This down-regulation affected the non-homologous end joining (NHEJ) pathway, an essential pathway for the repair of IR-induced DNA damage. The TNBC cells with the impaired NHEJ pathway did not recover from the damage caused by the IR and presented diminished survival rates [58]. Additionally, the ectopic expression of miR-200c affected the DNA repair mechanism by downregulating the expression of the lncRNA *LINC02582*, which in turn, downregulated USP7 and CHK1 expression, rendering the RR-TNBC cells more sensitive to IR [43]. It is well known, that lncRNAs (and circRNAs) can act as miRNA sponges, competing for miRNA target binding and reducing their regulatory effect. This mechanism can impact different biological processes, including the ones modulating radioresponse [59, 60].

In the additional studies investigating the effect of ncRNAs in apoptosis, caspase-3 emerged as the most targeted apoptotic protein. Four ncRNAs, miR-27a, miR-129-5p, miR-634, and lncRNA *CCAT1*, were identified conferring sensitivity to the irradiated cells by up-regulating caspase-3 expression, and consequently promoting apoptosis [28, 42, 52, 61]. Other molecules such as the anti-apoptotic BCL2 and PARP proteins, a marker for apoptosis known to suppress DNA repair, were also targeted. Overexpression of miR-31 [62] and miR-3188 [63] led to a decrease of *BCL2* and PARP expression protein levels, resulting in increased apoptosis and decreased cell viability, respectively, and enhancing the radiosensitivity in TNBC cells. MiR-93-5p also was described modulating radiosensitivity when overexpressed, by increasing apoptosis, inhibiting cell viability and migration in in vitro TNBC models [29]. The miR-93-5p action on this pathway that involves an Eph receptor tyrosine kinase and the transcription factor NF-κB, was evidenced by the reduction of tumor growth in the TNBC xenografts. Notably, miR-93-5p has been previously associated with deregulated expression in plasma exosomes from patients with breast cancer, in association with radiosensitivity [55]. Collectively these studies indicate the promising potential of miR-93-5p as a valuable marker for radioresponse.

Other ncRNAs were described to modulate radiosensitivity by their down-regulated expression levels. For instance, the downregulation of Circ_0008500 was shown to improve radiosensitivity and inhibit tumorigenesis in the irradiated TNBC cell line MDA-MB-468 through the miR-758-3p/PFN2 axis. This regulation led to the increase of BAX expression, a pro-apoptotic protein, and decreased expression of *BCL2* [34]. Moreover, the knockdown of *LINC00511* promoted apoptosis and increased the expression of *STXBP4* (STXBP4) levels through competitive binding to miR-185 [53].

In addition to the induction of apoptosis, the ncRNAs also act on autophagy in TNBC cells to enhance sensitivity to IR. The upregulation of miR-129-5p reduced the expression of autophagy-related proteins, such as HMGB1, LC3-II, and p62, which in turn decreased autophagy on MDA-MB-231 irradiated cells [42]. Conversely, miR-199a-5p regulated the autophagy induced by IR. The positive regulation of this miRNA maintained low levels of the autophagy-associated proteins LC3-I, LC3-II, and low levels of DRAM1 and Beclin1 expression, resulting in a controlled IR-induced autophagy rate in TNBC cell lines [49].

Restraint of DNA damage repair has been identified as a strategic approach to enhance radiosensitivity. In general, the induction of cell death through IR requires the accumulation of a substantial DNA damage, particularly DSB. However, tumor cells can take advantage of alternative molecular mechanisms that activate and drive the DNA repair processes. The activation of DNA damage repair (DDR) cascades may reduce the effectiveness of IR, ultimately promoting cell survival. Notably, several studies have shown that the negative regulation of critical DNA repair pathways can increase radiosensitivity [64–66]. The knockdown of *SIRT1* induced by miR-22 upregulation was demonstrated to restrain the DDR on MDA-MB-231 irradiated cell line, leading to a decrease in cell proliferation [40]. Moreover, miR-139-5p and miR-205 were associated with DDR pathways by targeting markers of these pathways. The ectopic transfection of miR-139-5p, causing a delay in DNA repair, promoted with double power the radiosensitivity potency compared to the presence of two or more DDR mutations [67]. For miR-205, its overexpression led to the downregulation of the expression of *ATM*, *ZEB1*, and *UBE2N* (*UBC13*), which code for proteins that act in the homologous recombination (HR)-mediated DNA damage repair pathway, as evaluated in the study of Zhang et al. [50] by the γ-H2AX assay. The inhibition of the HR pathway by this miRNA, enhanced the radiosensitivity of the TNBC cells [50]. MiR-142-3p and miR-302a were also reported to impact DNA repair by downregulating *BRCA1*/*BRCA2* and *RAD52* expression, which code for proteins involved in the HR pathway [51, 68].

In addition to the cellular processes cited above, the ncRNAs have been shown to modulate radiosensitivity by exerting control over the proliferation of stem cells populations. Two studies suggested that one of the mechanisms by which IR modulation occurs in TNBC cells is based on the inhibition of stem cells proliferation and self-renewal ability [39, 57, 68]. For example, let-7d downregulated the CyclinD1/Akt/Wnt1 pathway, resulting in a diminished stem cell population. Other targets related to stemness, such as *BOD1* and *KLF4*, were downregulated by miR-142-3p [39, 68]. Conversely, lower levels of the lncRNA *NEAT1* were found to be correlated with decreased stem cell renewal. The knockdown of this lncRNA, using the CRISPR-Cas9 method in a RR-TNBC cell line, led to the downregulation of key stemness genes, such as *BMI1*, *OCT4* and *SOX2*, resulting in decreased stem cell renewal and enhanced radiosensitivity [57].

The EGFR signaling pathway was another pathway affected by ncRNAs in the modulation of radiosensitivity. The ectopic expression miR-7 reduced the expression levels of *EGFR*, *AKT*, *ERK*, and *STAT3* and radiosensitized TNBC cells [69]. MiR-302a and miR-3188 were also reported to increase radiosensitivity of the TNBC cells by affecting the EGFR pathway, causing the downregulating of *AKT* expression [51, 63]. Similarly, miR-218 exerted a comparable action, but it targeted the primary downstream effector of EGFR, the p44/42 MAPK (ERK 1/2), decreasing cell survival upon IR exposure [44].

Finally, another described mechanism demonstrated to modulate radiosensitivity in TNBC was associated with the high-density lipoprotein (HDL). Wolfe et al. [41] showed that miR-33a negatively regulated HDL and induced radiosensitivity in TNBC cell lines composed of both inflammatory and non-inflammatory cells. These types of cells are present in several TNBC cell lines and are characterized by distinct expression patterns and clinical behavior [41]. In the cells expressing high levels of miR-33a, the transfection with anti-miR-33a decreased radioresistance, as evidenced by the reduction of colony formation in the clonogenic assays. Conversely, in cells with low levels of miR-33a, its ectopic expression reversed the HDL-induced radiosensitization. Notably, in BC patients treated with radiation, high miR-33a expression was associated with worse overall survival. This study highlights the importance of comprehensively characterizing the molecular signature and clinical characteristics of the distinct TNBC cell populations, as these differences may ultimately impact on the clinical prognosis of patients submitted to RdT protocols.

The data presented above is illustrated in Fig. 2 and described in Table 2.

#### NcRNAs modulating radioresistance

NcRNAs were also observed to confer radioresistance to the TNBC cells. However, a smaller number of ncRNAs were described compared to those that increased radiosensitivity (Table 3). Among these ncRNAs, one miRNA (miR-27a) and one lncRNA (*LINC00963*) were common to both groups. Their mechanisms of action were reversed; the inhibition of miR-27a enhanced radiosensitivity by increasing the expression of CDC27 and caspase-3 [28]. In contrast, its overexpression induced radioresistance by downregulating caspase-3 and BAX expression and by upregulating BCL2 [70]. For the *LINC00963*, Zhang et al. demonstrated that its downregulation induced IR sensitivity on TNBC [31], while Wang et al. showed the opposite effect [56]. The latter was attributed to the activation of the transcription factor *FOSB* and the increased of UBE3C protein expression, leading to the downregulation of *TP73* expression. The occurrence of these alterations increased cell proliferation and viability, and decreased apoptosis [56].

As for other miRNAs involved in radioresistance, including miR-144 [71], miR-454a [72], and miR-1290 [45], their overexpression was shown to be associated with the increase of cell viability and reduction of apoptosis. MiR-1290, specifically, acted by reducing the expression of pyroptosis-related markers, such as IL-18, IL-1β, ACS, and caspase-1 [45].

Finally, for the lncRNA, *HOTAIR*, the increased radioresistance was attributed to its role as a sponge to miR-499b-5p [33]. This action resulted in a decrease of miR-499b-5p expression levels, preventing its negative regulatory effect on *HSPA1A*. This, in turn, led to the indirect increase of *HSPA1A* levels, which conferred higher tolerance to IR in the TNBC in vitro and in vivo models evaluated [33].

The data presented above is illustrated in Fig. 2 and described in Table 3.

### NcRNAs as potential clinical biomarkers and therapeutic targets

In addition to describing the roles of the ncRNAs that confer both radioresistance and radiosensitivity in TNBC cell models, 11 of the 37 selected articles reported their potential use as biomarkers of tumor response to IR and as therapeutic targets (Table 4). Essentially, the results based on the expression levels of distinct ncRNAs among the cell lines pre- and post-exposure to IR and/or non-radioresistant (wild type) and radioresistant (RR) cell lines and/or non-resistant and resistant TNBC tissues and/or liquid biopsies, highlighted them as potential biomarkers for monitoring IR response.

**Table 4:** NcRNAs as potential clinical biomarkers of radiotherapy response in TNBC

ncRNA	TNBC samples	Expression levels pre-IR/post-IR*	Refs.
*MiRNAs*			
*miR-122*	*RR-*MDA-MB-231 *WT*-MDA-MB-231 + 4 Gy	Down/Up Down/Up	[48]
*miR-199a-5p*	*WT*–MDA-MB-231 + IR	Up/Down	[49]
*miR-205*	*RR*–SUM159-P2	Up/Down	[50]
*miR-302a*	*WT*–MDA-MB-231, SKBR3 + 0, 4, 8 Gy *RR*–MDA-MB-231	Up/Down Up/Down	[51]
*miR-634*	*RR*–MDA-MB-231	Up/Down	[52]
*miR-1290*	*RR*–MDA-MB-231 *Radioresistant*–TNBC Tissues	Down/Up _	[45]
*LncRNAs*			
*AFAP1-AS1*	*Radioresistant* – TNBC Tissues *RR-*MDA-MB-231 *RR-*MDA-MB-231	Down/Up Down/Up	[32]
*CCAT1*	*WT*–MDA-MB-231 + 2 Gy	–/Up	[61]
*NEAT1*	*RR*–MDA-MB-231	–/Up	[57]
*CircRNAs*			
*Circ_0008500*	*WT*–MDA-MB-468 + 4 Gy	Up/Down	[34]
*CircNCOR1*	*WT*–MDA-MB-231 + 6 Gy	Up/Down	[54]

*NcRNA,* Non-coding RNA; *TNBC,* Triple-negative breast cancer; *IR,* Ionizing radiation; *Up,* upregulated; *Down,* downregulated; –, information not available; *Ref,* reference; *Gy,* grays; *WT,* Wild type; *RR,* Radioresistant. Expression levels evaluated by RT-qPCR, Human Apoptosis miScript miRNA PCR Array, LncRNA microarray or RNA sequencing

Overall, seven articles [32, 34, 48, 49, 51, 54, 61] evaluated ncRNAs as early predictors of TNBC response to RdT, including three miRNAs (miR-122, miR-199-5p, and miR-302a), two lncRNAs (*AFAP1*-*AS1* and *CCAT1*) and two circRNA (Circ_0008500 and CircNCOR1). Other studies, including some mentioned above, showed ncRNAs as prognostic markers of radioresistance in TNBC; among them five miRNAs (miR-122, miR-205, miR-302a, miR-634, and miR-1290) and two lncRNAs (*AFAP1*-*AS1* and *NEAT1*) [32, 45, 48, 50–52, 57].

MiR-122 expression levels were observed increased in the MDA-MB-231 cells after exposure to 4 Gy compared to the parental cell line. The same pattern was observed in the constructed RR-MDA-MB-231 cell line. These findings suggested that miR-122 could be used as an early response marker for predicting radioresistance [48]. On the other hand, miR-199a-5p was found to be negatively regulated by irradiation [49]. Similar results were observed for miR-302a in MDA-MB-231 cells irradiated with different doses of IR [51]. Interestingly in this article, not only miR-302a, but all members of the miR-302 family, presented reduced expression after IR exposure. Further, miR-302a expression levels in RR-MDA-MB-231 were decreased compared to the parental cells. [51]. Decreased expression of miR-634 was also observed in RR-MDA-MB-231 compared to non-resistant cells in the study of Yang et al. [52]. On the other hand, miR-205 showed decreased expression in the radioresistant cell line SUM159-P2 compared to the parental cells [50]. Finally, miR-1290 expression was evaluated in clinical samples (serum and tumor tissues) and described with higher expression in patients that present with radioresistance compared to those with radiosensitivity [45]. These findings, together with the previous study describing overexpression of miR-1290 in resistant TNBC cell lines, strongly suggested its role as a potential clinical biomarker as well as a novel therapeutic target for preventing TNBC radioresistance.

The lncRNAs described with roles as potential clinical and therapeutic markers were *AFAP1-AS1*, *CCAT1*, and *NEAT1*. Bi et al. (2020) conducted lncRNA microarray analysis in surgically resected tumors and core needle biopsies from TNBC patients before post-operative RdT and after post-operative RdT with local recurrence. These authors showed that the up-regulation of *AFAP1-AS1* induced radioresistance and was also upregulated on the RR-TNBC cell line. Furthermore, the evaluation of IR response in a TNBC xenograft model with a nano-s*iAFAP1-AS1*, showed decreased tumor growth under 10 Gy of irradiation, indicating the promising use of this nanoparticle as a druggable target for increasing TNBC response to IR [32].

The lncRNA *CCAT1* was found upregulated in irradiated (2 Gy) MDA-MB-231 compared to non-irradiated cells. Under the same irradiation conditions, this cell line showed a negative regulation of miR-148b, suggesting the potential of *CCAT1* and miR-148b as potential early-response biomarkers to IR [61]. Finally, the lncRNA *NEAT1* was found upregulated on RR-MDA-MB-231 compared to non-resistant MDA-MB-231 cells [57].

Two circRNAs described in association with response to IR, were the Circ_0008500 and CircNCOR1. Circ_0008500 levels were significantly decreased in MDA-MB-468 cells exposed to a 4 Gy radiation dose compared to non-irradiated cells [34]. In MDA-MB-231 cell line upon 6 Gy radiation, CircNCOR1 showed a decreased expression compared with the parental MDA-MB-231 cell line [54].

The ncRNAs described, their expression levels pre- and post-IR are presented in Table 4.

## Limitations of the studies and perspectives

The studies of this review highlight the advances in predicting RdT resistance and response to treatment based on ncRNAs. However, the translation of ncRNAs as reliable RdT biomarkers and the RNA-based therapies into the clinical practice requires extensive investigation. Some of the described studies provided comprehensive evaluations of the involved mechanisms by which the ncRNAs modulate radiotherapy, while others were more concise and did not offer in-depth experimental assays that could provide strong mechanistic evidence of the ncRNAs’ functional roles in radioresistance.

Additionally, the effective implementation of the developed RNA-base therapies requires rigorous testing of the immunogenicity, pharmaceutical and delivery cell properties. Despite their numerous advantages, such as high versatility and diverse functional repertoire, particularly attractive given the multifaceted nature of tumorigenesis and tumor heterogeneity [73–76], several technical and biological challenges must be addressed to ensure their successful clinical use [73, 76]. These factors should be carefully considered in both pre-clinical and clinical studies testing ncRNAs inhibitors for cancer treatment, particularly in advanced cases with refractory RdT responses [77].

## Conclusions

In conclusion, this comprehensive review shows the intricate and multiple involvement of ncRNAs in modulating the response to radiation in TNBC. The identified mechanisms of action attributed to these ncRNAs involved the modulation of cell survival, particularly through the regulation of autophagy and apoptosis, and DNA damage repair. The versatility of ncRNAs extends beyond their modulatory functions, positioning them as promising biomarkers for predicting treatment responses and attractive targets for therapeutic interventions. It is important to note that while the results derived from in vitro and in vivo assays provide valuable insights, the analysis of clinical samples, whether tissues or liquid biopsies, significantly elevates the robustness of the evidence. Undoubtedly, this marks an emerging field where the role of ncRNAs in radiation response in TNBC is gradually unraveling, holding the promise for the effective development of ncRNAs-based radiotherapy strategies.

## Data Availability

The datasets used and/or analysed during the current study are available from the corresponding author on reasonable request.

## Abbreviations

γ-H2AX: H2A.X Variant Histone protein-DNA damage marker
ACS: Pyroptosis marker
AFAP1-AS1: Actin filament-associated protein 1 antisense RNA1
ATM: ATM Serine/Threonine Kinase
BAX: BCL2 associated X, apoptosis regulator
BC: Breast cancer
BCL2: BCL2 apoptosis regulator
BMI1: BMI1 proto-oncogene, polycomb ring finger
BOD1: Biorientation of chromosomes in cell division 1
BRCA1: BRCA1 DNA repair associated
BRCA2: BRCA2 DNA repair associated;
Caspase-1/-3/-7/-9: Apoptosis-related cysteine peptidases
CCAT1: Colon-cancer-associated transcript-1
CDC27: Cell division cycle 27
CircNCOR1: Hsa_circ_0042174
CircRNAs: Circular RNAs
DDR: DNA damage repair
DNA: Deoxyribonucleic acid
DSB: Double-strand-breaks
DUXAP8: Double homeobox A pseudogene 8
DRAM1: DNA damage regulated autophagy modulator 1
EGFR: Epidermal growth factor receptor
ER: Estrogen receptor
ERK: Mitogen-Activated Protein Kinase 1
FOSB: FosB proto-oncogene
AP-1: Transcription factor subunit
Gy: Grays
HDL: High density lipoprotein
HER2: Human epidermal growth factor receptor-type 2
HMGB1: High mobility group box 1
HOTAIR: HOX transcript antisense RNA
HR: Homologous recombination DNA repair pathway
HSPA1A: Heat Shock Protein Family A (Hsp70) Member 1A
IL-1β: Interleukin 1 beta
IL-18: Interleukin 18
IR: Ionizing radiation
KLF4: KLF Transcription Factor 4
LC3: Microtubule-associated protein 1A/1B-light chain 3
LINC00511: Long intergenic noncoding RNA 00511
LINC00963: Long intergenic noncoding RNA 00963
LncRNAs: Long non-coding RNAs
MAPK: Mitogen-activated protein kinase
MiRNAs: MicroRNAs
NEAT1: Nuclear paraspeckle assembly transcript 1
NHEJ: Non-homologous end joining
NcRNAs: Non-coding RNAs
OCT4: Stem cell pluripotency and transcription factor Oct4
p62: Autophagy protein
PARP: Poly(ADP-Ribose) Polymerase
PCAT6: Prostate cancer associated transcript 6
PDL-1: Programmed cell death ligand 1
PFN2: Profilin 2
PR: Progesterone receptor
SSB: Single-Strand-Breaks
PRISMA: Preferred reporting items for systematic reviews and meta-analyses
QUIPS: Quality in prognosis studies
RAD52: RAD52 Homolog
DNA: Repair protein
RdT: Radiotherapy
RR: Radioresistant
RS: Radiosensitive
ROS: Reactive oxygen species
SIRT1: Sirtuin 1
SOX2: SRY-box transcription factor 2
STAT3: Signal transducer and activator of transcription 3
STXBP4: Syntaxin binding protein 4
TNBC: Triple-negative breast cancer
TP73: Tumor Protein P73
UBE2N: (UBC13)
UBE3C: Ubiquitin protein ligase E3C
UBC13: Ubiquitin E2 conjugating enzyme
UBQLN1: Ubiquilin 1
USP7: Ubiquitin specific peptidase 7
ZEB1: Zinc finger e-box binding homeobox 1
Wnt1: Wnt family member 1
